# The development and function of dendritic cell populations and their regulation by miRNAs

**DOI:** 10.1007/s13238-017-0398-2

**Published:** 2017-03-31

**Authors:** Haibo Zhou, Li Wu

**Affiliations:** Tsinghua-Peking Joint Center for Life Sciences, Tsinghua University School of Medicine, Institute of Immunology Tsinghua University, Beijing, 100084 China

**Keywords:** dendritic cell, mirna, activation, development, autoimmune disease

## Abstract

Dendritic cells (DCs) are important immune cells linking innate and adaptive immune responses. DCs encounter various self and non-self antigens present in the environment and induce different types of antigen specific adaptive immune responses. DCs can be classified into lymphoid tissue-resident DCs, migratory DCs, non-lymphoid resident DCs, and monocyte derived DCs (moDCs). Recent work has also established that DCs consist of developmentally and functionally distinct subsets that differentially regulate T lymphocyte function. The development of different DC subsets has been found to be regulated by a network of different cytokines and transcriptional factors. Moreover, the response of DC is tightly regulated to maintain the homeostasis of immune system. MicroRNAs (miRNAs) are an important class of cellular regulators that modulate gene expression and thereby influence cell fate and function. In the immune system, miRNAs act at checkpoints during hematopoietic development and cell subset differentiation, they modulate effector cell function, and are implicated in the maintenance of homeostasis. DCs are also regulated by miRNAs. In the past decade, much progress has been made to understand the role of miRNAs in regulating the development and function of DCs. In this review, we summarize the origin and distribution of different mouse DC subsets in both lymphoid and non-lymphoid tissues. The DC subsets identified in human are also described. Recent progress on the function of miRNAs in the development and activation of DCs and their functional relevance to autoimmune diseases are discussed.

## INTRODUCTION

DCs were discovered by Ralph Steinman and Zanvil Cohn in the late 1970s, while the notion that DCs have a unique role in the immune system was met with decades of skepticism (Steinman and Cohn, 1973; Steinman and Cohn, 1974). Forty years later, the exquisite ability of DCs to mount adaptive immune responses to foreign antigens is gradually recognized, and their contribution to the induction of tolerance to self-antigens is also becoming increasingly evident. Consequently, the potential therapeutic benefits of modulating the development and functions of DCs for vaccines against pathogens, tumors, and suppressive therapies for autoimmune diseases have attracted more attention in the research and clinical applications.

In the mid-1990s, the full scope and significance of DC diversity was first acknowledged. The finding that the DCs in murine spleen include two subsets defined by the presence or absence of CD8 expression and with distinct immune functions, substantially broadened our knowledge of the roles of DCs in the induction of adaptive immunity (Shortman and Heath, 2010). However, the existence of distinct DC subsets in non-lymphoid tissues was recognized much later. This was mainly due to the fact that although the functionally equivalent DC subsets shared several phenotypic features with their counterparts in lymphoid tissues, they did not, in fact, express CD8. Nevertheless, they were eventually characterized by the expression of the integrin CD103 (Helft et al., 2010).

Moreover, another major subset of the DC family was identified as a population of cells morphologically resembled plasma cells with weak antigen presentation ability, but, upon exposure to viral stimuli, they can produce large amounts of type-I interferon. In addition, upon viral stimulation these cells can also differentiate into immunogenic DCs that can prime T cells against viral antigens. These cells were named plasmacytoid DCs (pDCs) to distinguish them from the conventional DCs (cDCs) (Colonna et al., 2004).

MicroRNAs (miRNAs) are small regulatory non-coding RNAs that repress target transcripts post-transcriptionally. Within the past decade, the role of microRNAs (miRNAs) in immunology has been studied extensively. The importance of miRNAs in the control of differentiation and function of hematopoietic cells is clearly demonstrated by studies that genetically disrupt critical enzymes important for the biosynthesis of miRNAs (Xiao and Rajewsky, 2009). By gene targeting, it has been shown that genetic deficiency of Drosha results in the loss of mature miRNAs. In hematopoietic cells, T cell-specific Drosha and Dicer conditional knockout mice both spontaneously develop lymphoproliferative multi-organ inflammatory disease and die within a few weeks after birth (Chong et al., 2008). While the earliest studies of miRNA function in the immune system have demonstrated an essential role for miRNAs as a whole, later studies have focused on the contribution of specific miRNAs to specific immunologic processes. The important roles of miRNAs in hematopoietic development, cancer, immune homeostasis, inflammatory disease, and autoimmunity were gradually clarified. Such roles include their ability to negatively regulate signaling pathways and the expression of transcription factors essential in lineage commitment. It has been shown that development of specific cell lineages is often dependent on specific miRNAs. MiRNAs also play important roles in immune tolerance and many immune-related miRNAs regulate cell growth and apoptosis, leading to their high frequency of association with hematologic malignancy (Mehta and Baltimore, 2016). In this review, we will discuss the function of miRNAs in regulating DC differentiation and functions and their association with autoimmune diseases.

## DC IN LYMPHOID TISSUES

### DC in thymus

**Figure 1:**
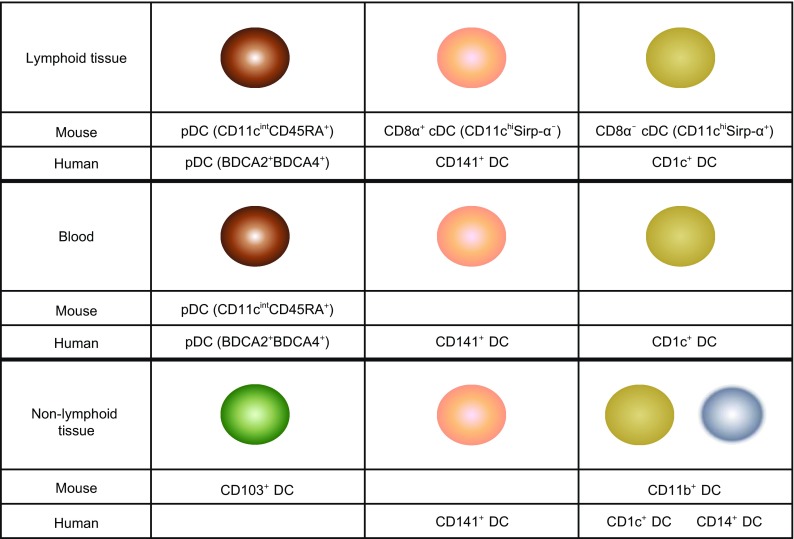
**DC subsets in the lymphoid and non-lymphoid tissues of mouse and human**. Lymphoid tissues in mouse contain pDC, CD8α^+^ cDC and CD4^+^ cDC, while lymphoid tissues in human contain pDC, CD141^+^ DC and CD1c^+^ DC. Mouse peripheral blood contains pDC while human blood contains pDC, CD141^+^ DC and CD1c^+^ DC. Non-lymphoid tissues in mouse contain CD103^+^ DC and CD11b^+^ DC, while non-lymphoid tissues in human contain CD141^+^ DC, CD1c^+^ DC and CD14^+^ DC

The thymus is a central immune organ and an important site for T cell differentiation, selection and generation of naive CD4^+^ and CD8^+^ T lymphocytes. Mouse thymus also contains CD11c^int^CD45RA^+^ plasmacytoid DCs (pDCs) and two CD11c^hi^CD45RA^−^ conventional DC (cDC) subsets that can be segregated on the basis of CD8α and the signal regulatory protein-α (Sirp-α) expression, as CD8α^+^Sirp-α^−^ and CD8α^−/lo^Sirp-α^+^ cDC subsets (Fig. 1) (Lahoud et al., 2006; Donskoy and Goldschneider, 2006). The CD8α^+^Sirp-α^−^ subset representing about 70% of thymic cDC is generated within the thymus from the earliest intrathymic progenitors, whereas the minor CD8α^−/lo^Sirp-α^+^ cDC subset originates from the peripheral migratory DCs (Donskoy and Goldschneider, 2006). Thymic cDC, although sharing many common features, differ from other peripheral DC subsets in that the majority of thymic cDCs in mouse is derived from an intrathymic precursor, and the former mostly present self-antigens (Ag) rather than foreign Ag (Wu et al., 1995; Wu et al., 1996). Thymic cDCs play important roles in the induction of central immune tolerance through a process called negative selection that deletes the developing thymocytes with self-reactivity and the generation of the naturally occurring CD4^+^CD25^+^ regulatory T cells (Goldschneider and Cone, 2003; Gallegos and Bevan, 2004; Watanabe et al., 2005; Bonasio et al., 2006; Proietto et al., 2008). Human thymus also contain DC subsets with similar functions as those found in the mouse (Bendriss-Vermare et al., 2001; Vandenabeele et al., 2001; Gurney et al., 2004; Keir et al., 2002).

### DC in spleen

The spleen is a rich source of lymphoid tissue-resident DCs. The DC populations in mouse spleen have been well characterized (Fig. 1). Three cDC subsets have been identified in the mouse spleen based on the surface expression of CD8α and CD4, in addition to high levels of CD11c expression on all cDCs. These cDC subsets are CD11c^hi^CD4^−^CD8α^+^, CD11c^hi^CD4^−^CD8α^−^, and CD11c^hi^CD4^+^CD8α^−^. The CD4^−^CD8α^+^ cDCs also express CD205, but not Sirp-α. In contrast, both CD4^−^CD8α^−^ and CD4^+^CD8α^−^ cDC subsets do not express CD205^−^, but are Sirp-α^+^, and are sometimes considered as one CD8α^−^ cDC subset (Lahoud et al., 2006; Vremec et al., 2000). The CD8α^−^CD205^−^ cDCs are located in the marginal zone, whereas the CD8α^+^CD205^+^ cDCs are in T cell areas. Marginal-zone DC can rapidly migrate into the T cell zone upon activation (De Smedt et al., 1996). A significant number of splenic cDC can be generated *in situ* by the intrasplenic immediate cDC precursors, named pre-DCs (Naik et al., 2006; Diao et al., 2006).

In addition to cDCs, pDCs are also found in mouse spleen. They are defined as CD11c^int^CD45RA^+^B220^+^SiglecH^+^. Similar to the blood pDC, the freshly isolated splenic pDC do not have the phenotypic and functional features of the antigen-presenting cDC, but can assume a cDC morphology and upregulate the cDC markers CD11c and MHC class II after activation with microbial stimuli. They represent the major cell type that produce large amounts of type-I interferon, a cytokine involved in innate immunity to virus. The pDCs in spleen migrate from the peripheral blood, because cells with the characteristics of pDC can be found in mouse blood, and the intrasplenic pre-DC do not differentiate into pDC (Asselin-Paturel et al., 2001; Nakano et al., 2001; O’Keeffe et al., 2002; O’Keeffe et al., 2003). Human spleen also contains pDCs, displaying plasma cell morphology, that selectively express Toll-like receptor (TLR)-7 and TLR9, and are specialized to rapidly secret massive amounts of type 1 interferon following viral stimulation. These are the CD4^+^CD11c^−^Lin^−^BDCA-2^+^BDCA-4^+^ cells (Siegal et al., 1999; Kadowaki et al., 2001; Liu, 2005; Mittag et al., 2011).

### DC in lymph node

The DC populations found in mouse LNs are more complex (Fig. 1). In addition to the three phenotypically and functionally equivalent cDC populations found in mouse spleen, two additional subpopulations have been described in the skin draining LNs. These correspond to the mature CD8α^lo^CD205^int^ and CD8α^lo^CD205^hi^ cDC that migrate from the epidermis and dermis, respectively, to the LNs. Subcutaneous LNs contain a higher percentage of the CD8α^lo^CD205^hi^ Langerhans cell (LC)-like cells than mesenteric LNs. The DCs derived from the migratory LC are responsible for carrying antigens picked up from skin to the draining LNs (Henri et al., 2001; Hochrein et al., 2001). In human LN, HLA^−^DR^+^CD11c^−^BDCA4^+^ cells have been identified as pDCs. HLA^−^DR^+^CD11c^+^ cells were separated into CD14^+^ and CD1a^+^ cells, which can be further divided into EpCAM^+^ LCs and CD1a^+^ DCs. CD1a^−^CD14^−^ cells can be further fractionated into Clec9A^+^ and BDCA1^+^ populations. Finally, BDCA1^+^ cells are comprised two subsets which either do or do not express CD206. Similar analysis of lymphoid organs that do not drain the skin showed that three of these DC subsets (LCs, CD1a^+^, and CD206^+^ DCs) were absent from cervical LNs draining the oropharynx, iliac LNs, tonsils, and spleen, suggesting that these DCs in skin-draining LNs are unique to and derived from the skin (Segura et al., 2012).

## ORIGINS OF LYMPHOID TISSUE DC

**Figure 2:**
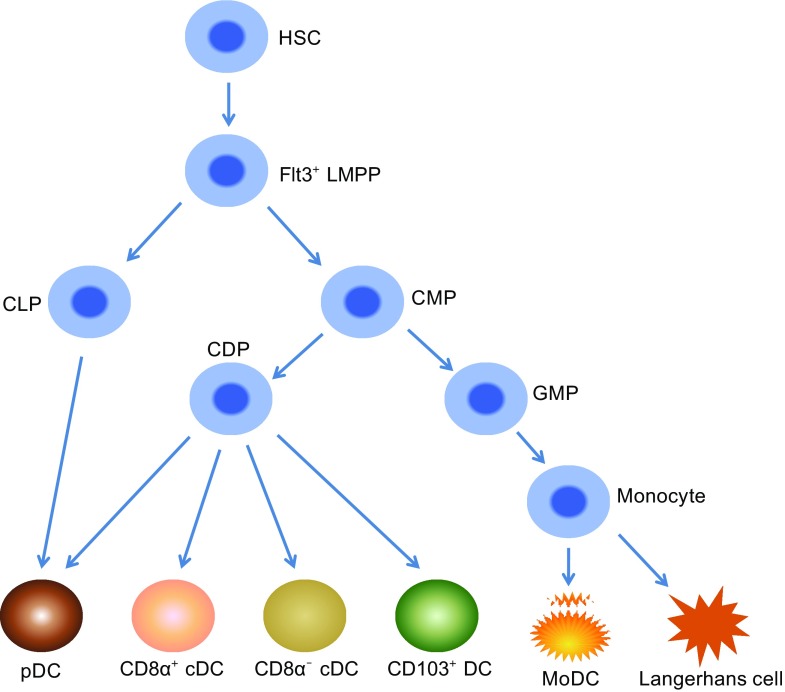
**The development of different DC subsets**. All DC subsets are derived from Flt3^+^ LMPP. CD8α^+^ cDC, CD8α^−^ cDC and CD103^+^ DC originate from CMP and CDP. pDC are differentiated from CLP, CMP and CDP. Langerhans cells and moDC are from monocytes

DCs, like all other leukocytes, develop from bone marrow-derived hematopoietic stem cells. Both cDC and pDC can be generated from the Flt3 expressing early myeloid or lymphoid progenitors, and Flt3L is essential for the development of steady-state DC populations (Fig. 2). When common lymphoid precursors (CLPs) and common myeloid precursors (CMPs) were purified from mouse bone marrow (BM) and adoptively transferred intravenously into irradiated recipient mice, they both showed the potential to give rise to splenic cDCs and pDCs. However, CMPs are 10-fold more abundant than CLPs; therefore, most spleen cDCs originate from CMPs. pDC are also derived from CMP, CLP, and DC restricted precursors CDP (common DC precursors) when these precursors are transferred into irradiated recipients (Wu et al., 2001; Manz et al., 2001; D’Amico and Wu, 2003; Martín et al., 2000).

## DC IN PERIPHERAL TISSUES

Non-lymphoid tissue-resident DCs are present in most tissues in the steady state (Fig. 1). Phenotypically, these DCs lack the expression of other hematopoietic cell lineage markers, but express the hematopoietic specific marker CD45, the integrin CD11c, and constitutively the major histocompatibility complex class II (MHCII). Several studies have now established that this definition is too broad and includes distinct DC populations with different origins and functions. Studies of non-lymphoid tissue DCs so far have largely involved tissues that are in contact with the body surfaces, such as the skin, lung, and intestine. We therefore will focus our review on the DCs from these specific tissues.

### DC in intestine

The intestine harbors a complex system of organized lymphoid tissues as primary sites for the induction of adaptive mucosal immune responses, including ileal Peyer’s patches, colonic isolated lymphoid follicles, and mesenteric lymph nodes (MLNs). Due to the difficulty of obtaining normal human gut tissues, most of the studies were done using mouse models. Like other tissue cDCs, mouse intestinal non-lymphoid tissue cDCs are Flt3L dependent and pre-DC derived (Bogunovic et al., 2009; Varol et al., 2009; Schulz et al., 2009; Edelson et al., 2010). Under steady-state, all intestinal cDCs are migratory cells, arising from classic DC precursors, without monocytic intermediates.

Intestinal DCs were originally defined as CD11c^+^ cells which were then subfractionated into intestinal macrophages (now best defined as CD11c^+^CD64^+^ cells) and DC (MHCII^+^CD11c^+^CD64^−^) subsets. MHCII^+^CD11c^+^ DCs included subpopulations best characterized as CD103^+^CD11b^−^ and CD103^+^CD11b^+^ (Bogunovic et al., 2009; Varol et al., 2009). The generation of intestinal CD103^+^CD11b^−^ cDCs depends on BATF3, Id2, and IRF8 and these cells thus represent the gut equivalent of splenic CD8α^+^ cDCs (Edelson et al., 2010; Klebanoff et al., 2013). Intestinal CD103^+^CD11b^+^ cDCs were subfractionated via the surface markers CD24 and CD64, revealing their composition of *bona fide* Flt3-dependent CD24^+^CD64^−^ cells and contaminating CD24^−^CD64^+^ cells that represent intestinal macrophages.

The development of CD24^+^CD103^+^CD11b^+^ DCs depends on transcription factor IRF4 (Persson et al., 2013), and the presence of granulocyte-macrophage colony stimulating factor (GM-CSF) is also required for the generation of CD103^+^CD11b^+^ lamina propria (LP) DCs (Farache et al., 2013). These CD103^+^CD11b^+^ LP DCs have been implicated in both the maintenance of tolerance towards the commensal microflora, and the generation of protective immune responses against pathogens (Jaensson et al., 2008; Farache et al., 2013). In the steady state, the functional properties of DCs appear to vary according to their anatomical locations. Indeed, CD103^+^CD11b^+^ DCs are required *in vivo* for efficient generation of Treg cells (Scott et al., 2014; Spadoni et al., 2012; Goto et al., 2014).

### DC in lung

The lung, like the intestine, is vulnerable to pathogenic insult and is constantly exposed to potentially harmful substances. Studies have highlighted the abundance of DCs in lung tissue and their dynamic accumulation upon stimulation. The mouse lung is grossly divided into large conducting airways and lung interstitium containing alveolar septa and capillaries where gas exchange is taking place. Under steady-state conditions, the conducting airways of all species studied are lined with an intraepithelial highly dendritic network of MHCII^hi^CD11c^hi^ cells that are mostly CD11b^−^ and express langerin and the mucosal integrin CD103 (αEβ7) (Lambrecht and Hammad, 2009).

In addition the network has the propensity of extending dendrites into the airway lumen through formation of tight junctions with bronchial epithelial cells. Immediately below, the lamina propria of the conducting airways contains MHCII^hi^CD11c^hi^ cells that also express high levels of CD11b, but not CD103, and are a rich source of proinflammatory chemokines (Lambrecht and Hammad, 2009; Guilliams et al., 2013; Grayson, 2006). The CD11b^+^CD103^−^ subset also expresses the signal regulatory protein-α (SIRPα) molecule. The exact location of the CD11c^int^ Siglec-H^+^ BSA-1^+^ pDCs in the lung is unclear although they can be found to line alveolar septa *in situ* and have been recovered from digests of large conducting airways. The alveolar space also contains CD11c^hi^MHCII^hi^ DCs and is easily accessible by broncho alveolar lavage.

The development of lung CD103^+^CD11b^−^ cDCs depends on Id2 and IRF8, aligning these cells with the classical CD8α^+^ and CD103^+^ DC lineage (Edelson et al., 2010; Ginhoux et al., 2009; Schlitzer et al., 2013). Consistent with this, lung CD103^+^CD11b^−^ cDCs are specialized in cross-presentation of antigens to CD8^+^ T cells. Notably, lung CD103^+^CD11b^−^ cDCs prominently express RALDH after Ag inhalation and facilitate *de novo* Treg cell induction (Plantinga et al., 2010). The lung CD103^−^CD11b^+^ cDCs are outnumbered by the cross-presenting CD103^+^ cDCs. More recently, CD11b^+^ cDCs, further defined as being CD24^+^, were shown to be dependent on IRF4. The CD11b^+^CD24^+^ cDCs were found to direct a Th17 cell response after challenge possibly due to their production of IL-23 (Schlitzer et al., 2013; Kim and Lee, 2014). Fate-mapping systems have also shown that while almost all lung CD103^+^ cDCs are CDP derived, this is not the case for CD11b^+^ cDCs, for which only about 50% of cells have been found to be of CDP origin (Liu and Nussenzweig, 2010). The additional developmental pathway for lung CD11b^+^ cDCs in the steady state remains to be defined.

### DC in skin

The skin can be divided into two anatomical sites: the epidermis and the dermis. The epidermis composed of keratinocytes is a tightly packed stratified epithelium that forms the water-impermeable stratum corneum. A basement membrane separates the epidermis from the underlying dermis, which is made up of fibroblasts and of intertwined collagen and elastin fibers (Merad et al., 2008; Malissen et al., 2014). The mononuclear phagocyte system is composed of DCs, monocytes, and macrophages. In the skin, DCs may include pDC, cDC, and moDC. Under steady-state conditions, pDCs are absent from the skin. They have only been identified in inflamed skin where they promote wound repair and mediate the systemic pro-inflammatory response that is seen after stimulation with Toll-like receptor 7 (TLR7) agonists (Gregorio et al., 2010).

Mouse cDCs in the dermis are generally categorized using the expression of CD11c and CD11b (also known as ITGAM). Dermal CD11c^+^CD11b^−^ cDCs express the C-type lectin langerin (also known as CD207 and CLEC4K) and include CD103^+^ and CD103^−^ cells (Poulin et al., 2007; Crozat et al., 2011). They are developmentally related to the CD8α^+^ cDCs that reside in secondary lymphoid tissues (Crozat et al., 2011; Hildner et al., 2008). Dermal CD11b^+^ cDCs are the most abundant type of DC in healthy dermis and, until recently, it has been difficult to distinguish them from CD11b^+^ monocyte- derived DCs and macrophages. The cDCs that are found in healthy non-lymphoid tissue, such as the skin, are not ‘at rest’. A small proportion of these cDCs undergoes a maturation process that is referred to as homeostatic maturation and that involves morphological and phenotypical changes that lead to their migration to the draining lymph nodes (Wilson et al., 2008). Such homeostatic maturation occurs continuously in germ-free mice and does not require TLR signals (Wilson et al., 2008).

Following homeostatic maturation, non-lymphoid tissue cDCs upregulate their expression of MHCII molecules and can transport cutaneous self-antigens to the T cell zones of the draining lymph nodes. If they encounter T cells that have escaped central tolerance, these cDCs trigger an abortive programme of activation in autoreactive T cells. In response to activation by protozoal, fungal, viral or bacterial stimuli, cDCs that reside in skin undergo a terminal differentiation program that differs from homeostatic maturation in that it additionally results in upregulation of the expression of co-stimulatory molecules. Such maturation leads to the migration of cDCs to draining lymph nodes, where they promote the clonal expansion of naïve antigen-specific T cells and the acquisition of T cell effector functions (Probst et al., 2003; Spörri and Reis e Sousa, 2005).

In human dermis, during steady-state conditions, DCs are classically divided into CD14^+^ and CD1a^+^ DCs (Nestle et al., 2009). CD14^+^ dermal dendritic cells (DDC) show variable expression of CD1a, CD1c, and CD163 (Haniffa et al., 2009). CD14^+^ DDC express low levels of CD80 and CD86 and are relatively poor inducers of naïve T-cell proliferation. They do however efficiently take up antigen, possibly due to expression of c-type lectins like CD206 and CD209/DC-SIGN (Haniffa et al., 2012; Klechevsky et al., 2008; Angel et al., 2007; Angel et al., 2006; Nestle et al., 1994). They also have the ability to induce Treg through high production of IL-10 (Chu et al., 2012). Skin-derived CD1a^+^ DCs in contrast express high levels of CD80 and CD86 and strongly induce allogeneic naïve CD4^+^ T cells and CD8^+^ T-cell proliferation (Haniffa et al., 2012; Klechevsky et al., 2008; Angel et al., 2007; Angel et al., 2006; Nestle et al., 1994). CD1a^+^ DDC isolated from skin-draining lymph nodes were found to preferentially induce TH2 polarization similar to LC (Segura et al., 2012).

## HUMAN DC SUBSETS IN PERIPHERAL BLOOD

In addition to the DC subsets described in different human tissues, human blood also contains different DC subsets. All blood DCs express high levels of HLA-DR and lack typical lineage markers CD3 (T cell), CD19/20 (B cell), and CD56 (natural killer cell). Myeloid DCs (mDCs) express typical myeloid antigens CD11c, CD13, CD33, and CD11b, corresponding to mouse CD11c ‘classical’ or ‘conventional’ DCs. In humans, both monocytes and mDCs express CD11c, but DCs lack CD14 or CD16 and can be split into CD1c^+^ and CD141^+^ subsets (Mittag et al., 2011; Jongbloed et al., 2010; Poulin et al., 2010; Crozat et al., 2010; Bachem et al., 2010), functionally corresponding to the mouse CD11b^+^ and CD8^+^/CD103^+^ cDC, respectively (Mittag et al., 2011; Jongbloed et al., 2010; Poulin et al., 2010; Crozat et al., 2010; Bachem et al., 2010). pDCs were first identified in humans. They typically lack myeloid antigens and can be distinguished by the expression of CD123, CD303, and CD304 (Cella et al., 1999; Mathan et al., 2013).

## INFLAMMATORY DC

During inflammation or infection, lymphoid and nonlymphoid organs can harbor DCs that originate from monocyte infiltrates and have been termed “monocyte-derived DCs” (moDCs) or “inflammatory DCs”. MoDCs are phenotypically difficult to discern from cDCs because they share similar expression patterns of MHC-II, CD11b, and CD11c. However, as an indicative of their monocyte precursor, moDCs express CD64, the Fc-gamma receptor 1 (FcγRI) (Mildner et al., 2013). Monocytes have long been known to give rise to DC-like cells that can efficiently stimulate T cells when cultured *in vitro* in the presence of GM-CSF and IL-4 (Plantinga et al., 2013). Gene expression profiles of cDCs and BM culture-derived DCs differ considerably. A relation of moDCs and cDCs is supported by the fact that culture-derived moDCs express the transcription factor zbtb46 that is restricted to cDCs present in the lymphoid tissues (Satpathy et al., 2012). MoDCs have since been described during pathogenic inflammation, experimental sterile inflammation, and in models of inflammatory diseases, such as colitis, rheumatoid arthritis (RA), systemic lupus erythematosus (SLE), and experimental autoimmune encephalomyelitis (EAE) (Ganguly et al., 2013).

## THE FUNCTION OF miRNA IN THE DEVELOPMENT OF DC

In DCs, CD11c-specific deletion of Dicer caused reduction in Langerhans cells without obvious perturbation of other DC subsets (Kuipers et al., 2010). However, within hematopoietic cells, miRNAs are differentially expressed by different lineages of hematopoietic cells and their precursors. Amongst DCs, the miRNA expression patterns are also distinct amongst different DC subsets (Kuipers et al., 2010).

It has been found that miR-22 is highly expressed in mouse CD11c^+^CD11b^+^B220^−^ cDCs compared to pDCs, and is induced in DC progenitor cell cultures with GM-CSF, which stimulates CD11c^+^CD11b^+^B220^−^ cDCs differentiation. Overexpression of miR-22 during DC development enhanced CD11c^+^CD11b^+^B220^−^ cDC generation at the expense of pDCs, while miR-22 knockdown demonstrated an opposite effect (Li et al., 2012). Overexpression and knockdown of miR-22 showed significant effects on the mRNA abundance of IRF8, a transcription factor essential for pDC and CD8α^+^ cDC development. These studies demonstrated that miR-22 was important in regulating the differentiation of DC subsets (Li et al., 2012).

Another important miRNA involved in DC homeostasis is miR-142. By analyzing miRNA expression profiles for distinct myeloid populations, including BM-resident progenitors, monocytes, and mature splenic DC subsets, miR-142 was found to be highly expressed in classic FLT3-L–dependent CD4^+^ cDCs, whereas reduced expression was observed in CD8α^+^ or CD4^−^CD8α^−^ cDCs (Mildner et al., 2013). Moreover, mice deficient for miR-142 displayed an impairment of CD4^+^ cDC homeostasis both *in vitro* and *in vivo*. Comparison of the expression profiles of WT and miR-142^−/−^CD4^+^ cDC equivalents using ingenuity pathway analysis revealed an up-regulation of the transcription factors HoxA9, IRF8, and Meis1 in miR-142^−/−^CD4^+^ cDCs. The up-regulation of IRF8 may suggest a function for miR-142 in the specification of CD4^+^ versus CD8α^+^ cDCs through regulation of the IRF8 pathway (Mildner et al., 2013).

## THE ROLE OF miRNA IN THE ACTIVATION OF DC

In addition to the role of miRNA in DC development, miRNA also functions in DC activation. When human moDCs were stimulated with LPS, miR-155 and several other miRNAs were highly up-regulated. Further *in vitro* studies showed that miR-155 acts as a positive regulator for the production of several pro-inflammatory cytokines including IL-6, IL-23, IL-12, and TNF-α by mouse moDCs stimulated using LPS. MiR-155 modulates cytokine production by targeting the negative regulators of signaling, such as SOCS1 and SHIP1 (Ceppi et al., 2009; O’Connell et al., 2009; O’Connell et al., 2010).

MiR-146a, which can also be induced by LPS stimulation, was found to negatively regulate DC activation. Knockdown of miR-146a caused an increase in NF-κB signaling through direct targeting TRAF6 and IRAK1 (Taganov et al., 2006). MiR-142-3p was also identified as a key negative regulator of IL-6. In contrast to miR-155, which is strongly up-regulated after LPS stimulation, miR-142-3p is constitutively and highly expressed in resting moDCs but down-regulated after LPS stimulation. MiR-142-3p directly targets IL-6 mRNA and thus specifically affects IL-6 expression (Sun et al., 2011).

Several miRNAs have been reported to modulate IL-12 production. MiR-21 was identified to directly inhibit IL-12p35 expression. Compared to wild type mice, *in vitro* derived moDCs from miR-21 deficient mice had enhanced production of IL-12, but not other cytokines (including TNF-α, IL-6, and IL-23) upon stimulation by LPS (Lu et al., 2011). MiR-10a also directly targets the IL-12 gene. Unlike miR-21, miR-10a negatively regulates the production of IL-12/IL-23p40. Ectopic expression of miR-10a in moDCs suppressed both production of IL-12 and IL-23 (Xue et al., 2011). Compared to miR-21 and miR-10a that directly target IL-12 genes, some other miRNAs target the signaling components that will affect multiple downstream targets. For example, miR-148/152 suppressed IL-12 as well as IL-6 production; miR-23b suppressed IL-12 production while enhancing IL-10 production (Liu et al., 2010).

Maturation of moDCs from human monocytes is accompanied by upregulation of DC-SIGN and downregulation of CD14. Based on DC-SIGN/CD14 expression ratios, miR-21, miR-34a, and their cognate targets WNT1 and JAG1 were found to negatively influence moDC differentiation (Hashimi et al., 2009). Similarly, inhibition of miR-511 and miR-99b resulted in reduced DC-SIGN levels (Tserel et al., 2011).

Although the main body of current research on the role of miRNAs in regulating DC differentiation and function used *in vitro* GM-CSF differentiated DCs, several studies have investigated other type of DCs. MiR-223 was found highly expressed in freshly isolated epidermal LCs, and lack of miR-223 significantly increased LCs-mediated antigen-specific CD8^+^ T cell proliferation *in vivo* and *in vitro* (Mi et al., 2013). pDCs are specialized cells that produce type-I IFN and express high levels of TLR7 and TLR9. Studies with human pDCs have revealed a role for miR-155 in fine tuning the TLR7-stimulated IFN-α production. Although TLR7 induced up-regulation of both miR-155* and miR-155 through the JNK pathway in pDCs, miR-155* induced during early phase of pDC activation enhanced IFN-α production by suppressing IRAKM, whereas miR-155 induced during later phase of pDC activation inhibited IFN-α expression by targeting TAB2. Thus, these two miRNAs cooperatively regulated the production of type-I IFN by human pDCs (Zhou et al., 2010).

## THE ROLE OF DC INTRINSIC miRNA IN AUTOIMMUNE DISEASE

The involvement of DCs in tolerance and autoimmunity is complex and bidirectional. Indeed, DCs may promote tolerance through multiple mechanisms, such as through central and peripheral tolerance induction, the generation and maintenance of Treg cells, as well as the induction of T cell unresponsiveness. Conversely, the powerful antigen presentation capacity of DCs may promote the activation and/or differentiation of self-reactive effector T cells, either because of the defective processes of central and peripheral tolerance induction or inappropriate activation of the self-reactive T cells due to impaired/ineffective negative regulation.

Increasing evidence has demonstrated that miRNAs are important regulators for normal immune responses and contribute to the prevention of autoimmunity (Table 1). They exert their effects either through regulating the differentiation and function of DCs or through direct regulation of immune responses.

**Table 1:** The role of miRNAs in autoimmune diseases

**miRNA**	**Predicted/Identified targets**	**Function**	**Related diseases**
miR-22	IRF8	Enhances CD11c^+^CD11b^+^B220^−^ cDC generation at the expense of pDCs	
miR-142	IRF8	Plays a pivotal role in the maintenance of CD4^+^ DCs	
miR-142-3p	IL-6	Specifically inhibits IL-6 expression by moDC	MS
miR-21	IL-12p35, Wnt1	Negatively regulates the production of IL-12 by moDC; negatively regulate the development of moDC	SLE, IBD, UC, MS
miR-10a	IL-12/IL-23p40	Suppress the production of IL-12 and IL-23 by moDC	SLE
miR-148/152	Calcium/Calmodulin- dependent protein kinase IIa	Suppress the production of IL-12 and IL-6	SLE
miR-23b	Notch1, NF-κB	Inhibits the production of IL-12 while promotes IL-10 production	UC
miR-155	SOCS1, SHIP1, TAB2	Positively regulates the production of several pro-inflammatory cytokines including IL-6, IL-23, IL-12, and TNF-α	RA, IBD
miR-146a	IRAK1, TRAF6	Negatively regulates TLR4-NF-κB pathway in monocytes	RA, SLE, IBD
miR-34a	JAG1	Negatively regulates the development of moDC	MS
miR-223	C/EBPβ	Negatively regulates LCs-mediated antigen-specific CD8^+^ T cell proliferation, production of inflammatory cytokine TNFα, IL-1β, and IL-23 by intestinal DCs. Positively regulates the differentiation of intestinal CX3CR1^+^ macrophages	UC
miR-29	IL-12p40, ATF2	Negatively regulates the production of IL-23 production by moDC	UC
miR-155*	IRAKM	Positively regulates the production of IFN-α by human pDC	

Rheumatoid arthritis (RA) is a systemic autoimmune disorder characterized by chronic inflammation of synovial tissue that results in irreversible joint damage. Inflammatory cytokines, especially TNF-α, IL-1β, and IL-6 are known to play an important role in RA pathogenesis, as inhibition of these cytokines can ameliorate disease in some patients (Smolen et al., 2007; Bresnihan et al., 1998). Stanczyk et al. reported increased miR-155 and miR-146a expression in RA synovial fibroblasts compared to those in osteoarthritis (OA) patients (Stanczyk et al., 2008). Furthermore, miR-155 expression was higher in RA synovial tissue compared to that of OA. MiR-155 expression was also higher in monocytes from RA synovial fluid compared to those from RA peripheral blood. Enhanced expression of miR-155 in RA synovial fibroblasts revealed matrix metalloproteinase 3 (MMP-3) as a potential target of miR-155, suggesting that miR-155 may modulate downstream tissue damage (Stanczyk et al., 2008). Since miR-155 is a positive regulator for moDC secreted IL-12p40, IL-12p35, and TNF-α (Dunand-Sauthier et al., 2011), the increased expression of miR-155 probably facilitates the excessive inflammatory response.

SLE is another systemic inflammatory autoimmune disease characterized by the presence of autoantibodies against numerous self-antigens including chromatin, ribonucleoproteins, and phospholipids. Seven miRNAs (miR-31, miR-95, miR-99a, miR-130b, miR-10a, miR-134, and miR-146a) were expressed at 6-fold lower level in SLE patients than that of healthy controls (Tang et al., 2009). Among these miRNAs, miR-146a in particular has been reported to negatively regulate the innate immune response by targeting interleukin-1 receptor–associated kinase 1 (IRAK1) and tumor necrosis factor receptor–associated factor 6 (TRAF6) in monocyte and moDC. Further analysis showed that under-expression of miR-146a negatively correlated with clinical disease activity and the interferon (IFN) scores in patients with SLE. Over-expression of miR-146a reduced, whereas inhibition of endogenous miR-146a increased, the expression of type I IFNs in peripheral blood mononuclear cells (PBMCs) (Steinman and Cohn, 1973). Besides, as miR-10a has also been demonstrated to negatively regulate DC function by direct targeting IL-12/IL-23p40 (Xue et al., 2011), its lower expression in SLE patients may also promote the autoimmune responses.

MiRNAs are also likely involved in the pathogenesis of psoriasis, a disease that may occur in association with inflammatory bowel disease (IBD). In a study comparing psoriasis patients to healthy controls, miR-203, miR-21, and miR-146a were significantly higher, while miR-123b was decreased in psoriatic skin compared to patients with atopic dermatitis and healthy controls. MiR-203 is expressed at the highest level in skin keratinocytes. One potential target of miR-203, SOCS-3, is upregulated in psoriatic lesions. Suppression of SOCS-3 activity leads to activation of STAT3, which when activated in keratinocytes in transgenic mice leads to the development of psoriasis (Sonkoly et al., 2007). Interestingly, a significant accumulation of FLT3^+^CD11c^+^ DCs in human psoriatic lesions and in the skin of experimental preclinical K14-VEGF transgenic homozygous mice, a mouse model for psoriasis. Targeted inhibition of FLT3 almost completely cured the psoriasis-like disease (Yan et al., 2014). As STAT3 is the key transcription factor for FLT3 induced DC production, the increased miR-203 expression may lead to activation of STAT3, and then facilitate pDC and cDC differentiation.

As seen in RA, SLE, and psoriasis, there is increasing data that patients with ulcerative colitis (UC) and Crohn’s disease (CD) have altered miRNA profiles in involved tissues compared to controls. The first study examined miRNA expression in IBD compared sigmoid colon biopsies from patients with active UC, inactive UC, chronic active CD, irritable bowel syndrome, and microscopic colitis with healthy control subjects. Eight miRNAs (miR-16, miR-21, miR-23a, miR-24, miR-29a, miR-126, miR-195, and Let-7f) were significantly increased in active UC tissues and three miRNAs (miR-192, miR-375, and miR-422b) were significantly decreased in the UC tissues compared to healthy controls (Wu et al., 2008). MiR-192 and miR-21 were the most highly expressed miRNAs associated with active UC in human colon tissues. MiR-21 has been demonstrated positively regulating the progression of DSS induced colitis and miR-21 deficient mice showed improved survival rate during DSS induction (Shi et al., 2013). A second study confirmed that miR-21 was elevated in inflamed tissue from 12 UC patients compared to 12 healthy controls. This study also identified upregulated miR-155 in inflamed tissues (Takagi et al., 2010). Similar to miR-21, miR-155 knockout mice showed alleviated symptoms in DSS induced colitis model (Singh et al., 2014). A third analysis of miRNAs in UC tissue examined eight UC patients and 10 healthy controls. Biopsies were obtained from inflamed and non-inflamed tissues of UC patients. In this study, five miRNAs (miR-29a, miR-29b, miR-126*, miR-127-3p, miR324-3p) were found upregulated and four (miR-188-5p, miR-25, miR-320a, miR-346) downregulated in both quiescent and active UC compared to healthy controls (Fasseu et al., 2010). As DCs are important regulators for IBD, we examined miRNA expression specifically in intestinal DC subsets and found that the expression of miR-223 by intestinal DCs decreased continuously during the progression of colitis. By using the DSS-induced colitis mouse model, we demonstrated that miR-223 deficiency resulted in more severe symptoms. Intestinal DCs in miR-223 deficient mice showed a more pro-inflammatory phenotype and a decreased number of CX3CR1^+^ regulatory macrophages was also observed in miR-223 deficient mice. Mechanistic study revealed that miR-223 play an important role in maintaining the homeostasis of intestinal macrophages and DCs by directly targeting C/EBPβ (Zhou et al., 2015). Although different colitis related miRNAs have been reported, most of the expression profiles of these miRNAs were obtained form the tissue or a mixture of different cell types. In order to get a better understanding of the functions of these miRNAs, further analysis of their changes in different cell types is required. As one example, miR-29a was shown to be a negative regulator for colitis by directly inhibiting the production of IL-23 by moDCs (Brain et al., 2013), it was also found upregulated in active UC tissues (Fasseu et al., 2010).

## CONCLUSIONS

Rapid progress has been made in recent years in our understanding of the roles of miRNAs in regulating differentiation and function of DCs. These studies have helped us to better understand the biological significance of these miRNAs. Meanwhile, increasing numbers of studies have suggested that the dysregulated expression of miRNAs in DCs may be closely associated with different immune disorders. However, most of the current available evidence was mainly derived from *in vitro* studies with GM-CSF-differentiated DCs, the equivalents of moDCs that become abundant during inflammation. It remains to be confirmed whether these findings from *in vitro* studies can be applied to *in vivo* settings when very dynamic interaction between multiple types of immune cells and multiple components of microbes occur. Furthermore, complex DC networks consist of many DC subsets with shared and distinct functions. It remains largely unknown how miRNAs regulate the development and function of different DC subsets. Further studies are required to clarify the contribution of miRNAs to the diversity of DC subsets in lymphoid and non-lymphoid tissues, and to the development of DC related immune disorders.
